# Both novelty and expertise increase action observation network activity

**DOI:** 10.3389/fnhum.2013.00541

**Published:** 2013-09-13

**Authors:** Sook-Lei Liew, Tong Sheng, John L. Margetis, Lisa Aziz-Zadeh

**Affiliations:** ^1^Brain and Creativity Institute, University of Southern CaliforniaLos Angeles, CA, USA; ^2^Division of Occupational Science and Occupational Therapy, Herman Ostrow School of Dentistry, University of Southern CaliforniaLos Angeles, CA, USA; ^3^Neuroscience Graduate Program, University of Southern CaliforniaLos Angeles, CA, USA

**Keywords:** body representation, experience, shared circuits, action observation network, mirror neuron system

## Abstract

Our experiences with others affect how we perceive their actions. In particular, activity in bilateral premotor and parietal cortices during action observation, collectively known as the action observation network (AON), is modulated by one's expertise with the observed actions or individuals. However, conflicting reports suggest that AON activity is greatest both for familiar and unfamiliar actions. The current study examines the effects of different types and amounts of experience (e.g., visual, interpersonal, personal) on AON activation. fMRI was used to scan 16 healthy participants without prior experience with individuals with amputations (novices), 11 experienced occupational therapists (OTs) who had varying amounts of experience with individuals with amputations, and one individual born with below-elbow residual limbs (participant CJ), as they viewed video clips of goal-matched actions performed by an individual with residual limbs and by an individual with hands. Participants were given increased visual exposure to actions performed by both effectors midway through the scanning procedure. Novices demonstrated a large AON response to the initial viewing of an individual with residual limbs compared to one with hands, but this signal was attenuated after they received visual exposure to both effectors. In contrast, OTs, who had moderate familiarity with residual limbs, demonstrated a lower AON response upon initial viewing—similar to novices after they received visual exposure. At the other extreme, CJ, who has extreme familiarity with residual limbs both visually and motorically, shows a largely increased left-lateralized AON response, exceeding that of novices and experienced OTs, when viewing the residual limb compared to hand actions. These results suggest that a nuanced model of AON engagement is needed to explain how cases of both extreme experience (CJ) and extreme novelty (novices) can result in the greatest AON activity.

## Introduction

Observing others' actions elicits a neural response in one's own sensorimotor regions, particularly in the bilateral premotor and parietal cortices collectively referred to as the action observation network (AON; Buccino et al., 2001; Caspers et al., 2010). A common hypothesis is that, within the AON, action observation engages the putative mirror neuron system (MNS), motor neurons that fire both during the performance of an action and the observation of the same or similar actions (di Pellegrino et al., 1992; Gallese et al., 1996; Rizzolatti et al., 1996). Researchers hypothesize that this system may allow one to internally simulate observed actions within one's own sensorimotor system, thus providing an enriched “understanding” of the other person's actions and intentions (Iacoboni, 2005; Keysers and Gazzola, 2007; Thioux et al., 2008). However, since single cell recordings demonstrating direct “mirror” properties of neurons within these regions is not often feasible in experiments with human subjects, in the current manuscript, we choose to use the term “action observation network” or “AON” to refer to these motor regions in the premotor and parietal cortices that are active both when participants observe actions and when they perform actions. This overlap is detected at the voxel-level using functional magnetic resonance imaging (fMRI).

Recent research has demonstrated that we may activate our own motor representations both when we observe familiar actions within our own abilities (e.g., dancers watching their own form of dance; Calvo-Merino et al., 2005) and unfamiliar or impossible actions beyond our own abilities (e.g., observing an individual with amputations moving her residual limb or robots performing rigid-body, as opposed to more natural, human-like, actions; Cross et al., 2011; Liew et al., 2013). While a wealth of literature suggests that there is greater AON activity for familiar, compared to unfamiliar actions (Calvo-Merino et al., 2005, 2010; Cross et al., 2006; Kim et al., 2011; Diersch et al., 2013), a small but growing body of literature suggests that there is greater activity for unfamiliar, compared to familiar actions (Cross et al., 2011, 2013; Liew et al., 2011, 2013). The discrepancies across these experiments could be attributed to different types of stimuli being used, as the studies in which familiar actions generated greater AON activity used stimuli that could be both more familiar to the individual or more interesting (e.g., ballet dancers watching ballet may both have a greater motor representation for the actions and be more interested in the actions, than when watching capoeira (Calvo-Merino et al., 2005); same for archers, compared to non-archers, watching archery (Kim et al., 2011). In contrast, the latter studies used stimuli that, while unfamiliar, may also be more interesting than the familiar stimuli. For instance, Liew et al. (2011) used familiar and unfamiliar symbolic gestures, where the familiar gestures were routinely encountered in daily life and required little attention to interpret, but the unfamiliar gestures were extremely novel and required more effortful processing. Similarly, Cross et al. (2011) showed human vs. robotic dance, and it is possible that the robotic dance moves were more interesting to participants than the typical human movements. Given this, experience-dependent modulations of AON activity may be more complex than can fit into a simple linear relationship, with either “familiar” or “unfamiliar” groups resulting in greater AON activity. Cross et al. (2011) recently put forth a non-linear model of experience and the AON that suggests that instances of *both* extreme familiarity and extreme novelty may activate the AON the most. Here we set out to test this model, using different types and amounts of experience when participants observed an individual with limb differences compared to a typically-developed individual with hands.

The current investigation focused on the possible influence of real-life interactions, which are far more varied than the types of experience typically introduced in controlled experiments on action representation. We wished to examine whether real-life experiences would result in comparable patterns of neural activity compared to controlled laboratory-based experiences. To do this, we examined how different amounts and types of interpersonal experience with an individual with an unusual effector (the residual limb of a woman with bilateral amputations) modulated the neural response during action observation. Participants were asked to observe videos of an amputee performing actions with her residual limb and a typically-developed actress performing hand actions while we used fMRI to measure the blood-oxygen-level dependent (BOLD) signal, an indirect measure of brain activity. During the scanning paradigm, individuals first observed videos of both effectors performing goal-directed actions (pre-visual exposure), then were shown extended video clips of both effectors performing different goal-directed actions to provide greater visual experience with both effectors (visual exposure), and were scanned again when observing both effectors performing goal-directed actions, similar to the pre-visual exposure session (post-visual exposure). We recruited participants that fell into three different categories: (1) novice individuals without prior interactions with individuals with amputations (novices), (2) occupational therapists (OTs) or occupational therapy students with prior interactions with individuals with limb differences (OTs), and (3) in the case of one participant, an individual who had congenital limb differences himself (CJ).

Data on the novice group only, who had no personal experience with individuals with limb differences, was previously reported in a separate analysis, which focused on the role of novelty and visual experience introduced during the experiment (Liew et al., 2013). We include data from this cohort here for between-group comparisons to examine differences between individuals with different amounts and types of experience. Specifically, we were interested in the difference in AON activity between individuals without experience (novices, pre-visual exposure), individuals with controlled visual experience (novices, post-visual exposure), individuals with real-life interactions with amputees (experienced OTs), and an individual with personal, motor and visual experience due to being born below-elbow residual limbs himself (participant CJ). Experienced OTs had all worked with clients who had amputations in a rehabilitation context and helped them learn how to perform daily meaningful activities independently (e.g., dressing, bathing, grooming), thereby observing them perform functional, goal-directed actions with their residual limbs. Participant CJ was born with below-elbow limb differences, and was also studying as an occupational therapy student at the time of his participation in this study. We examined the neurophysiological response when each of these individuals observed residual limb vs. hand actions in our experiment for the first time and after they were given prolonged visual experience with each of the effectors.

Our primary goal here was to understand whether different amounts or types of experience with an individual who has physical differences from oneself modulates neural patterns of activity during action observation. While we provided all cohorts with the same experimental paradigm for consistency, we were primarily concerned with the between-group differences in initial response (before controlled visual exposure in the laboratory) to individuals with different bodies, as this comparison best captures how the three groups' experiences affect AON activity. We were secondarily interested in whether visual experience provided in the experiment to novice participants would produce similar results to prior real-life interactions as found in experienced OTs or extreme experience as found in participant CJ, upon their initial viewing of both effectors. While this approach is not as systematic as providing varying amounts of only one type of experience (e.g., providing all individuals with differing amounts of visual exposure in the laboratory), previous work has suggested that both real-life social interactions and controlled action observation experiences in laboratory settings engage common motor resonance regions (Hogeveen and Obhi, 2012). We further felt that the part of the value in examining whether varying types of experience with individuals who have physical differences could produce similar neural results is being able to suggest that people can use multiple means to increase their understanding of those different from themselves, beyond the laboratory setting.

Our previous results with novices only (Liew et al., 2013) suggested that novice viewers initially demonstrate a greater AON response to the novel residual limb as compared to a familiar effector, the hand. Interestingly, we also found that after a period of visual experience with individuals with residual limbs, this effect was no longer visible, and they showed similar AON responses to both effectors. Based on these results, we hypothesized that real-life interactions with individuals with different bodies from one's own should result in similar neural patterns to both usual and unusual effectors, such that experienced OTs would represent the residual limb actions similarly to the hand actions upon the initial viewing (that is, less activity for this contrast than novice participants upon the initial viewing). This prediction is partially supported by research demonstrating that sensorimotor experience can modulate the AON response (Press et al., 2007; Catmur et al., 2008; Catmur, 2012). Catmur et al. (2008) showed that when participants learned to associate observation of a finger movement in one direction with movement of their own finger in the opposite direction, they demonstrated greater counter-motor activity (e.g., activity in the muscle opposite to the muscle viewed). Similarly, Press et al. (2007) demonstrated that, if given proper training, participants could strengthen the association between their own motor movements and robotic stimuli, resulting in similar responses when observing both robotic and human actions after (but not before) training. We anticipated that experience with an individual with residual limbs, whether visual or interpersonal, might produce a similarly “trained” modulation of AON activity. However, in our case, we expected individuals to learn to pair an observed action beyond their motor abilities (e.g., residual limb actions) with an action within their motor abilities (e.g., goal-matched hand actions), resulting in an attenuated difference between the two effectors, similar to the results of Press et al. (2007). We thus anticipated that the experienced OTs during initial viewing would yield similar results to novices after visual experience.

However, we expected that AON activity would positively correlate with experience in the experienced OT group, with very experienced OTs showing greater AON activity than those with less experience. Thus, more familiarity with the residual limb would draw attention to nuances of actions made by this effector, and increased activity. Furthermore, because OTs with greater experience work with individuals with residual limbs more frequently, there may be an increased motivation or interest in studying the actions of such limbs, that might drive them to pay more attention to this type of effector. Previous research suggests that both greater expertise (Calvo-Merino et al., 2005; Cross et al., 2006) and increased selective attention (Bach et al., 2007; Chong et al., 2008, 2009) may increase the AON response. Finally, we anticipated that actual personal, kinematic experience with a body part, such as that found in CJ (who has similar but not identical effectors to those in displayed in our stimuli), would produce much greater AON activation when observing the residual limb than the hand. This may be due his personal ability to map the kinematics of the novel residual limb onto his own existing motor representations in greater detail, as motor experience may augment AON activity above and beyond visual experience alone (Calvo-Merino et al., 2010), or due to greater interest and attention in another individual with residual limbs, who has effectors that are similar to, but not identical, to his own. Thus, we hypothesized that these many diverse findings regarding different types and amounts of attention might be unified in this study design, and that they might fit with the Cross et al. (2011) U-shaped model of familiarity and AON activation. To summarize, we anticipated that novice participants (extreme novelty) and CJ (extreme familiarity) would demonstrate greater AON activity upon initial viewing than experienced OTs (moderate familiarity).

## Materials and methods

### Ethics statement

This study was approved by the University of Southern California Institutional Review Board and was performed in accordance with the 1964 Declaration of Helsinki.

### Participants

Sixteen healthy, typically developed novice participants (7 females, 9 males; mean ± *SD* = 24.8 ± 4.8 years), eleven healthy, typically developed occupational therapy participants (9 females, 2 males; mean ± *SD* = 33.9 ± 11.5 years), and one healthy participant who was born with bilateral below elbow amputations (male; age 22; referred to henceforth as participant CJ), were recruited to participate in this study. All novice participants had little to no experience with individuals with amputees (Novices). Occupational therapy participants all had moderate to extensive prior experience working with individuals with amputations (6 practicing OTs, 5 advanced occupational therapy doctoral students with prior fieldwork/work experience). The OT cohort had a range of experience from occasional experience with patients with amputations for at least several years, to over 20 years of experience working specifically with people with amputations, with most reporting weekly to daily contact with individuals with limb differences. CJ was also an occupational therapy doctoral student at the time of scanning, although reported limited time working with patients who had amputations. Amount of experience for all participants was briefly quantified during the initial screening and further elaborated upon with an extensive behavioral questionnaire after the fMRI scanning procedure. Detailed questions were not asked prior to the fMRI experiment to avoid biasing participants to the goal of the study. All participants were right-handed, had normal or corrected-to-normal vision, and were safe for MRI. Written informed consent was obtained from all participants before inclusion in the study.

### Stimuli and procedure

All experimental procedures and analyses were performed identically to a previously reported study (Liew et al., 2013) and reported here for ease of understanding.

#### Action observation runs

**Figure 1:**
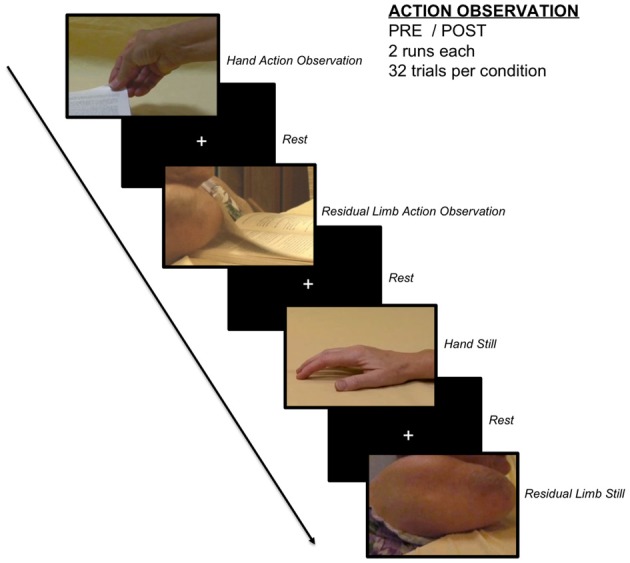
**Action observation paradigm**. Participants observed videos of goal-directed residual limb actions, goal-matched hand actions, still images of residual limbs, still images of hands, and a fixation cross in a randomized, event-related design.

Participants were shown 2-s videos of actions performed by an individual born without arms using her right upper residual limb (residual limb action observation, RLAO) and goal-matched actions performed by typically developed women using their right hands (hand action observation, HAO). Stimuli included videos of each actress using her effector to squeeze a binder clip, hit a ball, turn a book page, and press down the crease of a book. A depiction of the stimuli and basic scanner design can be found in Figure 1, and a schematic of the scanning procedure can be found in the Supplementary Information. While the experienced OT participants had a range of experience with individuals with residual limbs prior to the study, they had not viewed the particular individual with residual limbs depicted in the stimuli. Control stimuli used static images of each effector [hand still image (HS) and residual limb still image (RLS)], which were presented for 2 s each. To ensure participants were paying attention to the stimuli, 5 additional “catch” trials consisted of a red frame followed by an image of a hand. Participants were instructed to press a button on a button box when they observed these trials and told these trials were to ensure they paid careful attention to the stimuli. A fixation cross was presented during rest trials and jittered between 2 and 8 s in duration.

#### Visual exposure runs

To modulate experience during the scanning sessions, participants were presented with visual exposure to each effector in the form of 16-s blocks of short video clips of different actions performed by each effector. These videos provided more information about the body and context of each action, and included actions such as a hand and arm twisting the cap off of a water bottle or a residual limb manipulating a pencil.

#### AON localizer run

The AON is thought to respond both during the observation and performance of an action. Thus, a functional localizer run was used to identify neural regions were active both when participants observed and performed actions. Participants observed 3-s videos of a right hand picking up objects, action execution trials during which participants moved their right hand as though picking up a wine glass several times, still photos of a right hand next to an object without the connotation of movement, and rest trials of a black screen with a white fixation cross. Action execution trials were cued by a red box flashing briefly for 500 ms before a static image of a hand was presented for the remaining 2500 ms.

### Task design and procedure

Participants viewed 4 sample clips of all stimuli outside the scanner and were told this was in order to allow them to familiarize themselves with the stimuli and presentation types. In addition, however, we wanted participants to view sample clips of each effector in order to lessen any effects of initial shock or emotional reactions to seeing the novel effector for the first few times. In the case of experienced OT participants or CJ, we also felt that this allowed them an equal chance as novices to familiarize themselves with the specific effectors they would be observing. During the actual task, in action observation runs, participants were asked to watch the video clips that they saw and to pay attention to the movements and actions they saw. They were instructed to pay careful attention as at the end of each run, they might be asked, “What was the last action you saw?” to ensure they were paying attention. During the analyses, we grouped the first two action observation runs (PRE) and the second two action observation runs (POST) together, for a total of 32 trials per condition in each analysis.

After the first two action observation runs (PRE), we presented a visual exposure run, during which participants observed extended video clips (16 s each; 8 blocks per effector) consisting of each effector performing several actions in a row, followed by longer rest trials with a fixation cross (12 s). After this visual exposure run, participants observed another two action observation runs (POST). While we expected differences after visual exposure for the naïve subject group, we did not expect significant changes after visual exposure for the experienced OT group and participant CJ. Nevertheless, we kept the same experimental paradigm across subject groups for consistency.

Finally, participants were asked to complete an AON localizer run, during which they watched the hand actions and HS images on the screen, again paying attention to the movements and actions that they saw. They were also asked to move their right hand when they saw a red frame indicating an action execution trial. Once in the scanner, participants were monitored for extraneous movements via an MRI-safe mirror placed next to the scanner bed, which allowed for monitoring of hand movements from the control room. Non-task-related movements were monitored by an experimenter during the stimulus presentation, with no subject demonstrating non-task-related movement greater than 1% of trials.

After the scanning session, participants completed questionnaires about their familiarity with both residual limb and hand actions, and whether they felt watching the videos made them any more likely to interact with an individual with hands/residual limbs on a 10-point Likert scale (1 = very unfamiliar; unhelpful; unlikely to interact; 10 = very familiar; helpful; likely to interact; see Supplementary Information for a full list of questions), along with the Interpersonal Reactivity Index [IRI; (Davis, 1983)], a self-report behavioral measure of cognitive and emotional empathy.

### Scanning procedure

Images were presented through a projector onto a rear-projection screen attached to the head coil and located above the subject's head. The experiment utilized an event-related design in the Action Observation runs in which all conditions (HAO, RLAO, HS, RLS, rest) were evenly distributed across 4 runs, which lasted 336 s (168 TRs). The Visual Exposure run utilized a block design, counterbalanced across participants and lasted 464 s (232 TRs). The AON Localizer run utilized an event-related design lasting 492 s (246 TRs).

### Image acquisition

All images were acquired using a Siemens MAGNETOM Trio 3T MRI scanner with standard head coil. A high resolution T1-weighted anatomical volume was acquired from each participant (176 slices, 256 × 256 × 208 matrix with a spatial resolution of 1 × 1 × 1 mm, *TR* = 1950 ms, *TE* = 2.26 ms, *FOV* = 256 mm; flip angle = 9°). Functional volumes were acquired while participants performed the action observation, visual exposure, and AON localizer runs. Thirty-seven axial slices of functional images covering the whole brain were acquired using a gradient-echo echo-planar pulse sequence (64 × 64 ×37 matrix with a spatial resolution of 3.5 × 3.5 × 3.5 mm, *TR* = 2000 ms, *TE* = 30 ms, *FOV* = 224 mm, flip angle = 90°).

### Data processing and analyses

Functional data processing was carried out using FEAT (FMRI Expert Analysis Tool) Version 5.98, part of FSL (FMRIB's Software Library, www.fmrib.ox.ac.uk/fsl). The following pre-statistics processing were applied to individual subjects: motion correction using MCFLIRT (Jenkinson et al., 2002), slice-timing correction using Fourier-space time-series phase-shifting; non-brain removal using BET (Smith, 2002), spatial smoothing using a Gaussian kernel of FWHM 5 mm, grand-mean intensity normalization of the entire 4D dataset by a single multiplicative factor, and highpass temporal filtering [Gaussian-weighted least-squares straight line fitting, with sigma = 65.0; (Jenkinson et al., 2002; Smith, 2002)]. For each subject, a time-series statistical analysis was carried out using FILM GLM with local autocorrelation correction (MW Woolrich et al., 2001). Z (Gaussianised T/F) statistic images were then thresholded at *p* = 0.001 (uncorrected), and registered to a high resolution standard space image [2 × 2 × 2 mm^3^ Montreal Neurological Institute (MNI) space] using FLIRT (FSL's Linear Image Registration Tool); (Jenkinson and Smith, 2001; Jenkinson et al., 2002). A second-level analysis to average across the two runs in PRE and POST conditions, respectively, was carried out using a fixed effects model, by forcing the random effects variance to zero in FLAME [FMRIB's Local Analysis of Mixed Effects; (Beckmann et al., 2003; Woolrich et al., 2004; Woolrich, 2008)].

Group-level analyses were then completed using FLAME stage 1, which employed a mixed effects model that includes both fixed effects and random effects from cross session/subject variance (Beckmann et al., 2003; Woolrich et al., 2004; Woolrich, 2008). Z (Gaussianised T/F) statistic images at this level were thresholded using clusters determined by Z > 2.3 and a (corrected) cluster significance threshold of *P* = 0.05 (Worsley et al., 2001). Group analyses were run for novices and experienced OTs. A higher-level between-groups analysis was also run directly comparing novices to experienced OTs, with additional regressors of age and gender to account for these differences between groups. We compared novices and experienced OTs before visual exposure, after visual exposure, and most importantly, novices after visual exposure with experienced OTs before visual exposure. All results were thresholded at Z > 2.3 and a (corrected) cluster significance threshold of *P* = 0.05 (Worsley et al., 2001).

Region of interest analyses were also performed for *a priori* regions in the AON [bilateral inferior frontal gyrus/ventral premotor cortex and inferior parietal lobules (IFG/PMv and IPL)]. These four regions were defined by the overlap between action observation and action execution during the AON localizer run, and further masked by anatomical definitions based on the probabilistic Harvard-Oxford atlas of the IFG/PMv and IPL, respectively (results of which are shown in Liew et al., 2013). IFG and ventral premotor regions were combined into one region of interest as prior meta-analyses of the AON suggest that both comprise the frontal component of the AON (Van Overwalle and Baetens, 2009). Percent signal change (%SC) for the observation of each effector (HAO/RLAO) compared to the control still image (HS/RLS) was then extracted using Featquery in FSL. Correlation analyses between the %SC values from the ROI analyses and scores on the Interpersonal Reactivity Index and the demographics questionnaire, including experience with individuals with amputees, were also run in SPSS. Notably, experience with individuals with limb differences was only correlated with the experienced OT group. It was not correlated with ROI values in novices, as they did not have a wide enough range of experiences (all had minimal/no prior experience with individuals with limb differences). For additional ROI-based analyses, see Supplementary Information.

Data from participant CJ was analyzed as a single subject case and was also carried out using FEAT (FMRI Expert Analysis Tool) Version 5.98, part of FSL (FMRIB's Software Library, www.fmrib.ox.ac.uk/fsl). As CJ is only one participant, higher-level analyses were carried out using a fixed effects model, by forcing the random effects variance to zero in FLAME [FMRIB's Local Analysis of Mixed Effects; (Beckmann et al., 2003; Woolrich et al., 2004; Woolrich, 2008)]. Z (Gaussianised T/F) statistic images for CJ were still thresholded using clusters determined by Z > 2.3 and a (corrected) cluster significance threshold of *P* = 0.05 (Worsley et al., 2001) to provide a conservative estimate of effects. In order to visualize his single-subject results with both groups, we extracted ROI values from CJ and compared them with distribution in both novice and experienced OT groups using boxplots.

## Results

### Behavioral results

As expected, experienced OTs reported being significantly more familiar with residual limbs than the novice group (E: 7.18 ± 1.99; N: 1.31 ± 0.48; *t* = −9.58, *p* < 0.0001; TOTAL: 3.70 ± 3.21; CJ: 10). Experienced participants also reported that the videos helped them to understand residual limb actions significantly better they helped to understand than hand actions (RL: 5.64 ± 3.50, H: 4.73 ± 2.94, *t* = −2.65, *p* = 0.02) but unlike novices, they were not significantly more likely to interact with individuals with either effector after watching the videos (RL: 4.82 ± 3.12, H: 4.09 ± 3.33, *t* = −2.70, *p* = 0.12). Novices and experienced OTs did not significantly differ in their scores on the empathy measure (IRI TOTAL: N: 65.75 ± 9.67, E: 70.46 ± 6.59, *t* = −1.40, *p* = 0.17; IRI Perspective Taking: N: 19.75 ± 3.30, E: 19.00 ± 3.79, *t* = 0.55, *p* = 0.85; IRI Empathic Concern: N: 21.00 ± 3.76, E: 22.00 ± 2.886, *t* = −0.75, *p* = 0.30).

### Whole brain fMRI results: novices vs. experienced OTs

#### Novices vs. experienced OTs, action observation (PRE-visual exposure)

**Figure 2:**
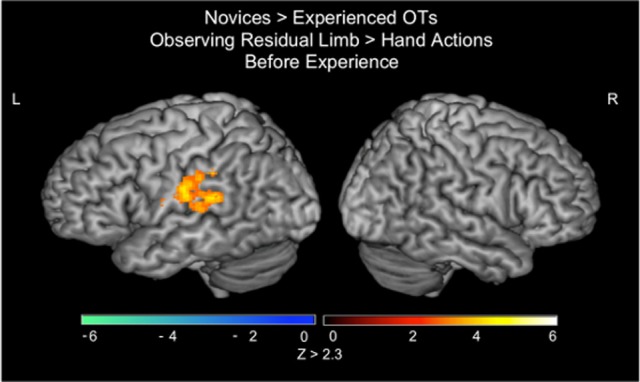
**fMRI results when novices view residual limb compared to hand actions for the first time compared to experienced occupational therapists**. Novices demonstrated greater activity in the left inferior parietal lobule during Residual Limb Action Observation > Hand Action Observation than experienced OTs. All results thresholded at *Z* > 2.3, *p* < 0.05 (cluster corrected for multiple comparisons).

In the PRE-visual exposure action observation runs, novices activated the left inferior parietal lobule (IPL) to a greater extent than experienced OTs when observing Residual Limb Action Observation compared to HAO (see Figure 2; Table 1). There was no significant activation that was greater for experienced OTs than novices in the PRE-visual exposure condition.

**Table 1:** **Localization of brain activations in novices vs. experienced occupational therapists**.

**Coordinates [*x y z*]**	**Anatomical region**	***Z*-stat**	**Cluster size [2 mm^3^ voxels]**	**Cluster index**
**RESIDUAL LIMB ACTION OBSERVATION > HAND ACTION OBSERVATION (PRE-VISUAL EXPOSURE)**				
[−38 −40 36]	L supramarginal gyrus (BA 40)	3.34	574	1

Group-level random effects analyses, thresholded at Z > 2.3, p < 0.05, cluster corrected for multiple comparisons. There were no suprathreshold clusters for contrasts of Hand Action Observation > Residual Limb Action Observation (PRE-visual exposure) or any of the POST-visual exposure contrasts.

#### Novices vs. experienced OTs, action observation (POST-visual exposure)

There was no significant activation for either novices greater than experienced OTs or the reverse in the POST-visual exposure condition.

### Novices (POST-visual exposure) vs. experienced OTs (PRE-visual exposure)

There were no significant activations for this contrast comparing Novices after visual exposure with experienced OTs before visual exposure.

### Whole brain fMRI results: novices

#### Novices, action observation (PRE-visual exposure)

**Figure 3:**
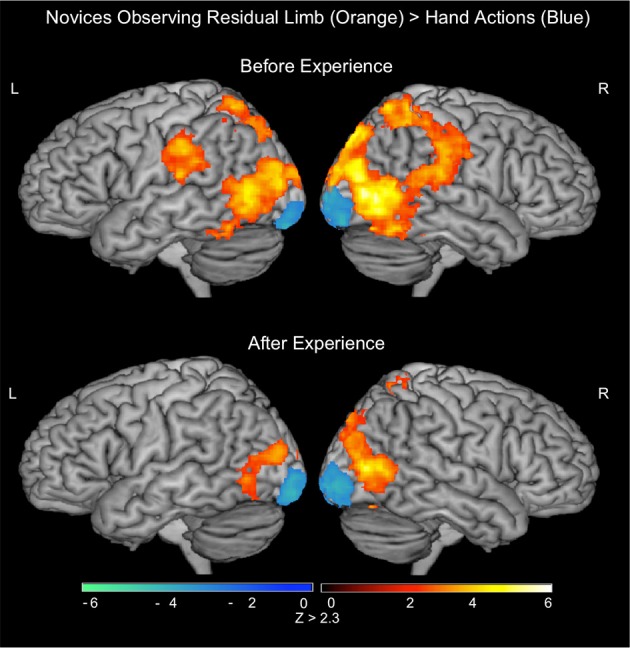
**fMRI results when novice viewers observe residual limb and hand actions for the first time (PRE novices) and after visual exposure (POST novices)**. TOP—PRE. ORANGE: Residual Limb > Hand activated sensorimotor regions, including the bilateral inferior and superior parietal lobules, and occipital regions. BLUE: Hand > Residual Limb generated significant activation in the bilateral occipital poles. BOTTOM—POST. ORANGE: Residual Limb > Hand activated the right superior parietal lobule and occipital regions, including MT/V5. BLUE: Hand > Residual Limb generated significant activation in the bilateral occipital poles. All results thresholded at *Z* > 2.3, *p* < 0.05 (cluster corrected for multiple comparisons).

During the PRE-visual exposure action observation runs, Residual Limb Action Observation vs. RLS images (RLAO > RLS) activated the right dorsal and ventral premotor cortices, the bilateral inferior and superior parietal lobules, and the bilateral lateral occipital cortices from the posterior middle temporal gyrus (MT/V5) into the medial lingual gyri (BA 17/18). HAO vs. HS images (HAO > HS) generated a similar pattern of activity in bilateral inferior and superior parietal lobules and the bilateral occipital cortices (MT/V5 into V1). In the direct contrast between Residual Limb and HAO (RLAO > HAO), there was activity in the bilateral IPL including the supramarginal gyrus, postcentral gyrus, and extending into the superior parietal lobules and the posterior middle temporal gyrus (MT/V5). Hand vs. Residual Limb Action Observation (HAO > RLAO) generated activity in the bilateral occipital poles (BA 17/18) only (see Figure 3; Table 2).

**Table 2:** **Localization of brain activations in novices during the PRE and POST conditions**.

**Coordinates [*x y z*]**	**Anatomical region**	***Z*-stat**	**Cluster size [2 mm^3^ voxels]**	**Cluster index**
**PRE-VISUAL EXPOSURE**				
**Residual Limb Action Observation > Still Photo of Residual Limb**				
[50 −66 0]	R lateral occipital cortex (MT/V5)	5.67	20946	3
[−48 −74 6]	L lateral occipital cortex (MT/V5)	5.62	–	3
[62 −34 22]	R inferior parietal lobule / supramarginal gyrus	5.11	–	3
[−58 −22 20]	L inferior parietal lobule / supramarginal gyrus	4.77	–	3
[34 −44 50]	R superior parietal lobule (BA 7)	4.58	1307	2
[52 6 38]	R ventral precentral gyrus	3.56	484	1
[44 2 48]	R dorsal precentral gyrus	3.54	–	1
**Hand Action Observation > Still Photo of Hand**				
[−50 −76 −2]	L lateral occipital cortex (MT/V5)	5.91	15589	3
[48 −60 0]	R lateral occipital cortex (MT/V5)	5.57	–	3
[66 −26 32]	R inferior parietal lobule	3.26	–	3
[−58 −24 22]	L inferior parietal lobule/supramarginal gyrus	3.95	936	2
[−58 −30 36]	L postcentral gyrus	3.55	–	2
[−48 −38 22]	L posterior superior temporal gyrus	3.40	–	2
[−32 −50 62]	L superior parietal lobule (BA 7)	3.96	344	1
**Residual Limb Action Observation > Hand Action Observation**				
[42 −70 2]	R lateral occipital cortex (MT/V5)	5.62	13686	3
[24 −60 −8]	R lingual gyrus (BA 17/18)	4.89	–	3
[62 −28 20]	R inferior parietal lobule	3.98	–	3
[44 −38 60]	R superior parietal lobule	3.01	–	3
[−50 −70 4]	L lateral occipital cortex (MT/V5)	4.45	5120	2
[−18 −60 64]	L superior parietal lobule	4.04	–	2
[−50 −30 34]	L inferior parietal lobule/supramarginal gyrus	4.12	1169	1
**Hand Action Observation > Residual Limb Action Observation**				
[28 −102 4]	R occipital pole	4.10	959	2
[−18 −94 −20]	L occipital pole	3.85	569	1
**POST-VISUAL EXPOSURE**				
**Residual Limb Action Observation > Still Photo of Residual Limb**				
[−46 −76 2]	L lateral occipital cortex (MT/V5)	5.30	15172	4
[44 −78 2]	R lateral occipital cortex (MT/V5)	5.23	–	4
[56 −30 18]	R inferior parietal lobule/supramarginal gyrus	3.94	–	4
[−44 −34 20]	L inferior parietal lobule/supramarginal gyrus	3.75	816	3
[34 −50 60]	R superior parietal lobule (BA 5/7)	4.09	765	2
[−34 −50 54]	L superior parietal lobule (BA 5/7)	3.68	422	1
**Hand Action Observation > Still Photo of Hand**				
[−46 −76 0]	L lateral occipital cortex (MT/V5)	5.81	19348	3
[46 −64 0]	R lateral occipital cortex (MT/V5)	5.22	–	3
[38 −56 54]	R superior parietal lobule (BA 7)	3.24	–	3
[−34 −52 56]	L superior parietal lobule (BA 5/7)	4.01	1100	2
[−40 −30 34]	L inferior parietal lobule/supramarginal gyrus	3.18	–	2
[60 −32 20]	R temporoparietal junction/posterior superior temporal gyrus	3.88	520	1
**Residual Limb Action Observation > Hand Action Observation**				
[50 −78 8]	R lateral occipital cortex (MT/V5)	4.90	4071	2
[22 −60 54]	R superior parietal lobule (BA 5/7)	3.37	–	2
[−36 −88 16]	L lateral occipital cortex (BA 18/19)	3.60	701	1
[−50 −68 −2]	L lateral occipital cortex (MT/V5)	3.43	–	1
**Hand Action Observation > Residual Limb Action Observation**				
[32 −94 −6]	R occipital pole	3.89	908	2
[−20 −98 −12]	L occipital pole	4.01	744	1

Group-level random effects analyses, thresholded at Z > 2.3, p < 0.05, cluster corrected for multiple comparisons.

#### Novices, action observation (POST-visual exposure)

After the Visual Exposure run, participants viewing Residual Limb Action Observation vs. RLS images (RLAO > RLS) activated the bilateral inferior and superior parietal lobules and bilateral occipital cortices from MT/V5 extending into V1, similar to the pre-visual exposure runs. HAO vs. HS images (HAO > HS) resulted in a similar pattern of activity with clusters of activity in bilateral superior parietal regions, left IPL, right posterior superior temporal sulcus (pSTS) into the IPL, and strong bilateral occipital activation (MT/V5 into V1). Residual Limb vs. HAO (RLAO > HAO) in the POST run demonstrated activity in the right superior parietal lobule and bilateral posterior middle temporal gyri (MT/V5). In contrast, Hand vs. Residual Limb Action Observation (HAO > RLAO) again generated activity in the bilateral occipital poles (BA 17/18) only (see Figure 3; Table 2).

### Whole brain fMRI results: experienced OTs

#### Experienced OTs, action observation (PRE-visual exposure)

**Figure 4:**
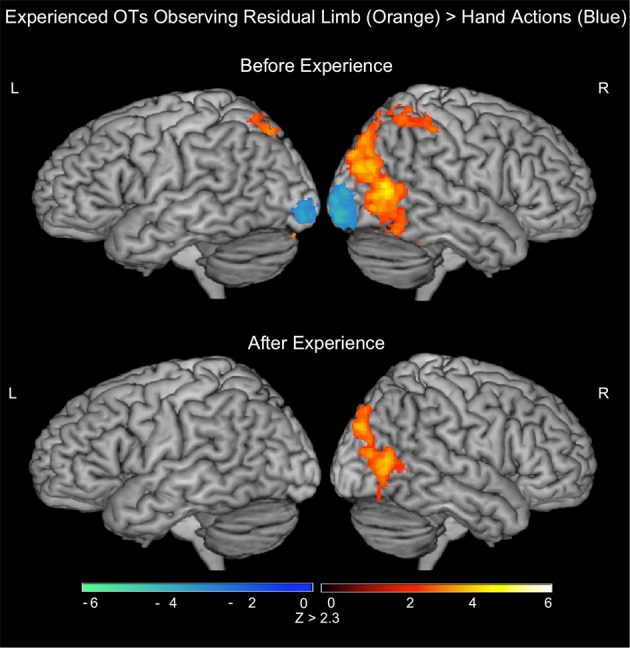
**fMRI results when experienced occupational therapists observe residual limb and hand actions for the first time (PRE experienced OTs) and after visual exposure (POST)**. TOP—PRE. ORANGE: Residual Limb > Hand activated the right superior parietal lobule and occipital regions. BLUE: Hand > Residual Limb generated significant activation in the bilateral occipital poles. BOTTOM—POST. ORANGE: Residual Limb > Hand activated the right occipital regions, including MT/V5. BLUE: Hand > Residual Limb did not generate any significant activation. All results thresholded at *Z* > 2.3, *p* < 0.05 (cluster corrected for multiple comparisons).

In the PRE-visual exposure action observation runs, experienced OTs observing Residual Limb Action Observation vs. RLS images (RLAO > RLS) activated the left inferior frontal gyrus, and bilateral premotor cortices, parietal cortices (inferior into superior lobules), and occipital cortices (MT/V5 into VI). HAO vs. HS images (HAO > HS) activated a similar pattern, with activation in the bilateral inferior frontal gyri and premotor cortices (dorsal and ventral), mid-anterior cingulate cortex, bilateral parietal cortices (inferior into superior, and bilateral occipital cortices (MT/V5 into V1). Comparing Residual Limb vs. HAO (RLAO > HAO) resulted in activity in the right posterior middle temporal gyri (MT/V5 into V1) and bilateral superior parietal lobules, with stronger activation on the right side, and in the left cerebellum. Hand vs. Residual Limb Action Observation (HAO > RLAO) generated activity in the bilateral occipital poles (BA 17/18) only. All results reported at the whole brain level (see Figure 4; Table 3).

**Table 3:** **Localization of brain activations in experienced occupational therapists during the PRE and POST conditions**.

**Coordinates [*x y z*]**	**Anatomical region**	***Z*-stat**	**Cluster size [2 mm^3^ voxels]**	**Cluster index**
**PRE-VISUAL EXPOSURE**				
**Residual Limb Action Observation > Still Photo of Residual Limb**				
[−46 −72 0]	L lateral occipital cortex (MT/V5)	4.68	19479	3
[58 −28 24]	R inferior parietal lobule/supramarginal gyrus	4.56	–	3
[52 −64 8]	R lateral occipital cortex (MT/V5)	4.40	–	3
[−54 −28 38]	L inferior parietal lobule/supramarginal gyrus	3.96	–	3
[−54 10 20]	L inferior frontal gyrus	4.25	855	2
[44 0 54]	R dorsal precentral gyrus	3.50	758	1
**Hand Action Observation > Still Photo of Hand**				
[−50 −70 10]	L lateral occipital cortex (MT/V5)	4.73	21784	3
[−56 −34 24]	L inferior parietal lobule/supramarginal gyrus	4.41	–	3
[58 −28 22]	R inferior parietal lobule/supramarginal gyrus	4.37	–	3
[54 −64 −2]	R lateral occipital cortex (MT/V5)	4.35	–	3
[48 6 14]	R inferior frontal gyrus	3.39	–	3
[−52 8 18]	L inferior frontal gyrus	4.13	2231	2
[2 −2 38]	R mid-anterior cingulate cortex	3.83	1840	1
**Residual Limb Action Observation > Hand Action Observation**				
[4 −82 −2]	R lingual gyrus (BA 17/18)	4.88	6406	2
[44 −66 8]	R lateral occipital cortex (MT/V5)	4.38	–	2
[31 35 61]	R superior parietal lobule (BA 7/19)	3.14	–	2
[−26 −72 –36]	L cerebellum	2.82	–	2
[−18 −74 54]	L superior parietal lobule (BA 7/19)	3.36	344	1
**Hand Action Observation > Residual Limb Action Observation**				
[32 −98 −8]	R occipital pole	4.11	1150	2
[−32 −94 −10]	L occipital pole	3.54	425	1
**POST-VISUAL EXPOSURE**				
**Residual Limb Action Observation > Still Photo of Residual Limb**				
[−50 −72 10]	L lateral occipital cortex (MT/V5)	4.81	5254	4
[52 −72 8]	R lateral occipital cortex (MT/V5)	4.81	3036	3
[30 −48 64]	R superior parietal lobule (BA 7)	3.89	687	2
[−58 −28 44]	L inferior parietal lobule/supramarginal gyrus	3.58	325	1
**Hand Action Observation > Still Photo of Hand**				
[50 −70 0]	R lateral occipital cortex (MT/V5)	5.02	4816	2
[−46 −70 0]	L lateral occipital cortex (MT/V5)	4.24	1611	1
**Residual Limb Action Observation > Hand Action Observation**				
[20 −70 −12]	R fusiform gyrus (BA 17/18)	4.41	2173	2
[46 −68 8]	R lateral occipital cortex (MT/V5)	3.95	–	2
[24 −86 32]	R superior lateral occipital cortex	3.84	495	1

Group-level random effects analyses, thresholded at *Z* > 2.3, *p* < 0.05, cluster corrected for multiple comparisons.

#### Experienced OTs—action observation (POST-visual exposure)

After the visual exposure run, experienced viewers observing Residual Limb Action Observation vs. RLS images (RLAO > RLS) activated the left IPL, right superior parietal lobule, and bilateral lateral occipital cortices (MT/V5). HAO vs. HS images (HAO > HS) generated activity in the bilateral occipital cortices, from MT/V5 into V1. Residual Limb vs. HAO (RLAO > HAO) in the POST run demonstrated activity in the right occipital cortex, from MT/V5 into V1 and into the superior lateral occipital cortex corresponding to V3. In contrast, Hand vs. Residual Limb Action Observation (HAO > RLAO) generated no significant activity. All results reported at the whole brain level. (see Figure 4; Table 3).

### Correlations

**Figure 5:**
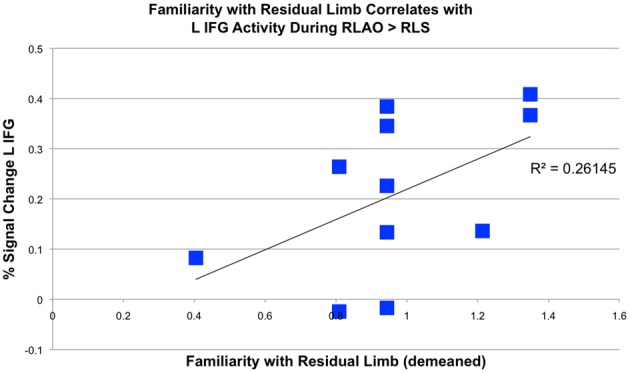
**Correlation between percent signal change in left inferior frontal gyrus during residual limb observation and familiarity with residual limbs in experienced OTs**. Percent signal change in the L IFG when experienced OTs observed Residual Limb Action Observation > Residual Limb Still (RLAO > RLS) in the PRE condition correlated with familiarity with the residual limb (*R*^2^ = 0.26, *p* = 0.05, 2-tailed).

Experienced OT participants reported being more familiar with residual limb actions than novices, with experienced OTs demonstrating a range of experience. In this group, familiarity with the residual limb correlated with activity in the L IFG ROI during residual limb action observation compared to RLS (*R*^2^ = 0.26, *p* = 0.05, 2-tailed; see Figure 5). However, experienced OTs did not show any significant correlations with empathy, which *were* seen in novice participants (Liew et al., 2013).

### Whole-brain fMRI results: participant CJ

#### CJ, action observation (PRE-visual exposure)

**Figure 6:**
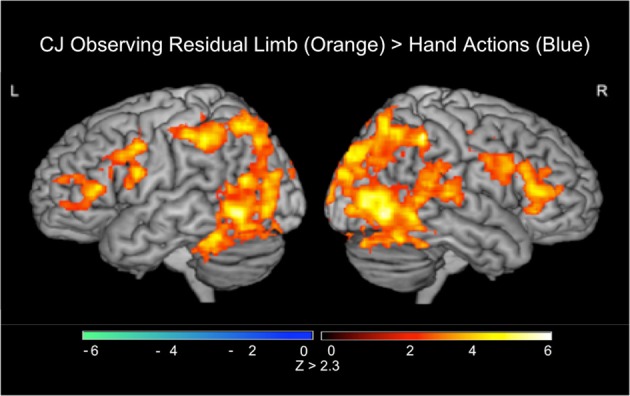
**fMRI results when CJ observed residual limb and hand actions (PRE)**. Residual Limb > Hand activated the bilateral inferior frontal gyri, premotor cortices, inferior and posterior parietal lobules, and occipital regions. All results thresholded at *Z* > 2.3, *p* < 0.05 (cluster corrected for multiple comparisons).

In the PRE-visual exposure action observation runs, observation of Residual Limb Action Observation vs. RLS images (RLAO > RLS) activated the right inferior frontal gyrus extending into the bilateral dorsal and ventral premotor cortices, the left ventral premotor cortex, and bilateral parietal cortices (inferior into superior lobules), along with bilateral occipital cortices (MT/V5 into VI). HAO vs. HS images (HAO > HS) activated only bilateral occipital cortices (MT/V5 into V1), although at a lower threshold, similar regions of bilateral premotor and parietal regions were active. Comparing Residual Limb vs. HAO (RLAO > HAO) resulted in activity in the bilateral inferior frontal gyri into the bilateral premotor cortices, bilateral inferior and superior parietal lobules, with stronger activation on the right side, the midline precuneus, and in the bilateral occipital regions (MT/V5 into V1) (see Figure 6). Hand vs. Residual Limb Action Observation (HAO > RLAO) was not associated with any significant activations. All results reported at the whole brain level.

#### CJ—action observation (POST-visual exposure)

After the visual exposure run, CJ observing Residual Limb Action Observation vs. RLS images (RLAO > RLS) activated the left IPL, right superior parietal lobule, and bilateral lateral occipital cortices (MT/V5). HAO vs. HS images (HAO > HS) generated activity in the bilateral occipital cortices, from MT/V5 into V1. Residual Limb vs. HAO (RLAO > HAO) in the POST run demonstrated a small cluster of activity in the right occipital cortex, roughly corresponding to the occipital fusiform gyrus, extending into the left hemisphere. In contrast, Hand vs. Residual Limb Action Observation (HAO > RLAO) generated no significant activity. All results are reported at the whole brain level.

#### CJ vs. novice and experienced OT groups

**Figure 7:**
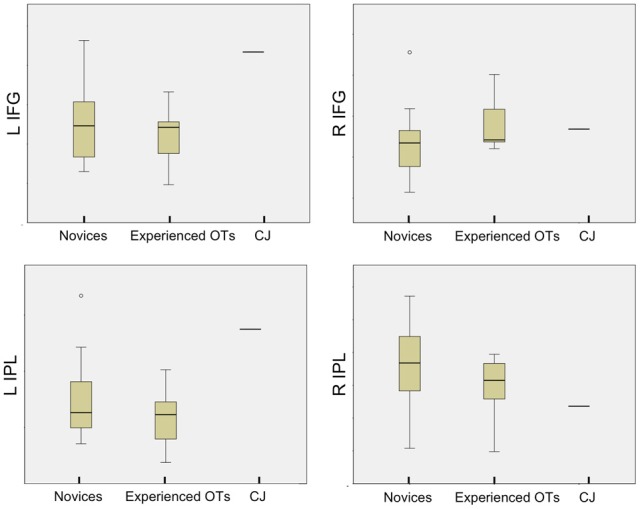
**Comparison of novices, experienced OTs, and CJ means across AON regions of interest**. Boxplots of novices, experience OTs and CJ mean percent signal change at each AON region of interest (L IFG, L IPL (on the left of the image), R IFG, R IPL (on the right of the image) during Residual Limb > Hand Action Observation, demonstrating that CJ's activation is higher than the group mean for novice and experienced OT groups at the L IFG and L IPL.

CJ demonstrated greater activation than the median activity in either novice or experienced OT groups during observation of the residual limb compared to hand in the left IFG and IPL (see Figure 7). His activity was similar to that of the novice participants and experienced OTs, in the right-hemisphere regions of interest (right IPL, right IFG).

## Discussion

The current study examined how different amounts and types of experience with an effector modulate activity within the AON when observing others' actions. In this study, participants with varying amounts of real-life interactions with individuals with limb differences (novices, experienced OTs) and an individual with limb differences himself (CJ) observed goal-matched actions performed by an individual with residual limbs and an individual with hands. Our results demonstrate that participants with moderate amounts of experience (OTs upon initial viewing and novice participants after receiving visual experience) demonstrated similar AON responses, with no significant differences between groups when observing residual limb vs. hand actions. In contrast, novice participants upon initial viewing (extreme novelty) and the individual with extreme experience (CJ, who has limb differences himself) upon initial viewing, demonstrated greater AON activity when observing residual limbs than hands. A positive correlation between the more experienced OTs with increased AON activation also suggests that as individuals gain significantly more experience, they generate greater activity in the AON. These results tie together prior conflicting studies by demonstrating that cases of both extreme experience and extreme novice may activate the AON more strongly than moderate amounts of experience. These results fit the recently proposed U-shaped model of experience in modulating the AON (Cross et al., 2011), discussed in detail below, and suggest that the AON is flexibly engaged both to generate new models of actions we have not seen before and cannot perform and to recall existing motor repertoires from actions we have seen, practiced, or performed extensively before.

### Different types of experience similarly attenuate the sensorimotor response to novel effectors

Novice participants upon their initial viewing (without experience) demonstrated greater AON activity than experienced OTs upon their initial viewing (with prior real-life interactions), particularly in the left IPL. This finding is in line with prior studies demonstrating greater activity when observing novel, compared to familiar, actions (Cross et al., 2011; Liew et al., 2011), such as novel gestures or robotic actions. The IPL plays a key role in extracting affordances for an effector and spatially mapping and encoding the kinematics of an effector onto one's own motor system (Rizzolatti et al., 1997; Oztop et al., 2006), and likely allows novice participants to match the kinematics of the novel effector onto their own existing motor repertoires (Liew et al., 2013). This IPL activation may be less necessary for experienced OTs, who already have an existing motor repertoire for the residual limb actions. After visual exposure, there was no difference between activation patterns for novice participants or experienced OTs, suggesting that the visual exposure for novices and the prior real-life interactions, plus visual exposure, for OTs attenuated the response to residual limb actions in both groups. For both experienced OTs and CJ, post-visual exposure runs resulted in attenuated AON activity, as seen also in novices.

Experienced OTs demonstrated similar neural responses to both hand and residual limb actions upon the initial viewing, suggesting that they represent both effectors similarly after having real-life interactions with individuals who have both. Importantly, this pattern of activation is similar to that of novices after they receive visual exposure, with both groups showing activation in the right superior parietal lobule and visual regions only when contrasting residual limbs vs. hand actions after their respective forms of experience (experimentally-introduced visual experience for novices, and real-life interactions for OTs). The fact that many different types of experience yield similar results supports previous findings showing that both visual and motor experience can induce similar AON responses (Cross et al., 2009) and both laboratory and real-life experiences can result in similar effects (Hogeveen and Obhi, 2012). In support of this, there were no significant differences between the two groups (novices with experimentally-introduced visual experience; experienced OTs with real-life interactions) when compared directly. It is likely that the right superior parietal activation supports participants' ability to update their pre-existing internal models of residual limb actions (Wolpert et al., 1998), which they generated either through visual (novices) or real-life (OTs) experience. This supports the hypothesis that moderate experience attenuates the difference between how we represent bodies similar to our own vs. those dissimilar to our own, and suggests that this applies across different types or forms of experience.

In addition to the right superior parietal lobule, experienced OTs observing residual limb compared to hand actions additionally activated the lateral posterior middle temporal gyrus (MT/V5), correlating with the putative extrastriate body area [EBA; (Downing et al., 2001)], and extending into the pSTS, an area often found active in with regions of the AON during action observation (Keysers and Gazzola, 2007; Engel et al., 2008; Liew et al., 2011). Both area MT/V5 and the pSTS have reciprocal connections with the parietal cortex to support spatial awareness and are particularly active in response to observed biological movements (Perrett et al., 1989, 1990; Seltzer and Pandya, 1989, 1994; Downing et al., 2001). Thus, it is not surprising that experienced OTs and novice participants after experience also activate these regions during observation of the novel residual limb compared to hands, as the residual limb may require more visual attention to the specific movement kinematics of the novel limb.

### Increased frontal AON activity correlates with familiarity in experienced OTs

The frontal component of the AON, the IFG, is thought to be involved with understanding the goals and intentions of an action (Iacoboni et al., 2005; Van Overwalle, 2009). In the current study we find that experienced OTs demonstrated a marginally-significant positive correlation between activity in the left IFG and the amount of experience they had with residual limb actions. This suggests that frontal AON activity may be increased with experience. The link between increased IFG activation and experience may thus be related to the experienced OT's ability to process the goals and intentions of individuals with residual limbs. Notably, OTs are specialized in examining how individuals perform goal-directed actions, which may affect these results. While the current correlation is weak, possibly due to the limited sample size, future research may test these findings in larger populations and may also consider including individuals who have different professions and different relationships with individuals with residual limbs to provide a more diverse and generalizable population sample.

### Both extreme novices and extreme experts show increases in sensorimotor activity

Finally, a case study of an individual (CJ) born with bilateral below elbow amputations provides more information about how experience—in many ways, extreme experience—affects sensorimotor activation when observing someone with a different body. As someone with a different body himself, CJ demonstrates extensive activation in all AON regions (bilateral inferior frontal, premotor, and parietal regions) when observing residual limb compared to hand actions. This activation is similar to novices upon their initial viewing of the stimuli but includes the bilateral premotor and parietal regions. From the boxplot, it is clear that CJ activates his left IFG and left IPL more than the mean of either novices or experienced OTs. This may be due to the dominance of the left hemisphere in object-directed actions (Vingerhoets et al., 2012), something that CJ may have greater experience with give his personal motor experience with residual limb movements.

**Figure 8:**
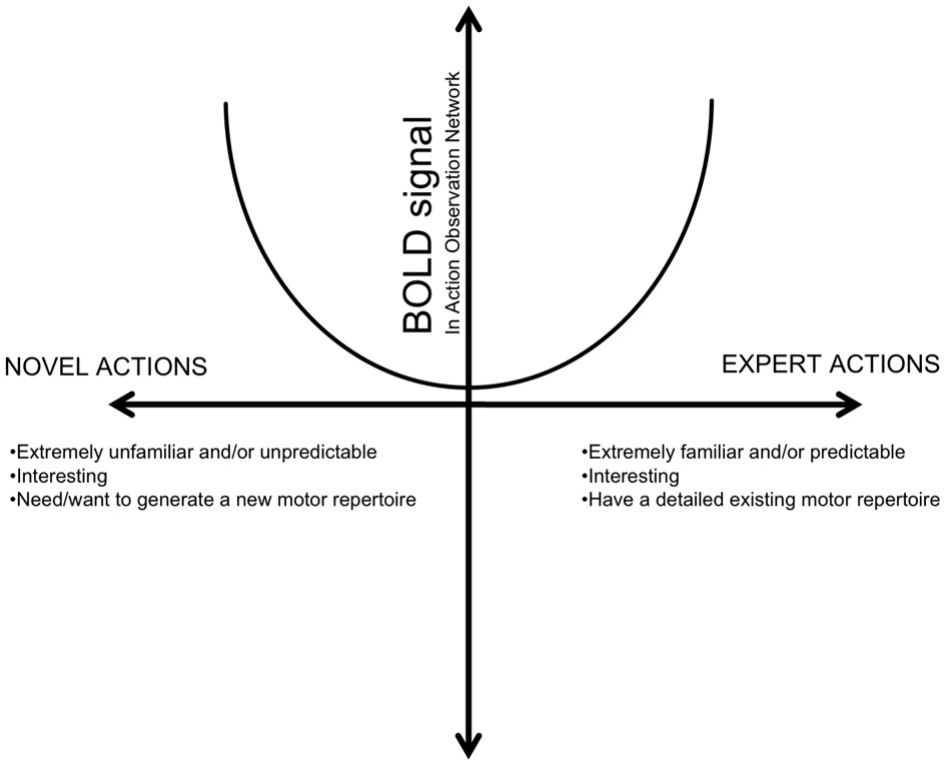
**A hypothesized relationship between BOLD response in the AON and action familiarity**. (Adapted from Cross et al., 2011, Figure 7). This relationship proposes that the AON is modulated by different types of familiarity, one's inherent motivation, and interest, among other factors.

In addition, this pattern is in line with our previous finding that, as OTs gain more experience, there is more activity in the IFG. However, in CJ, this pattern extends to other components of the AON as well. This is congruent with prior literature showing that extreme familiarity, such as in the case of expert dancers, generates greater AON activation and falls in line with the U-shaped model of experience recently proposed by Cross et al. (2011). In this model (adapted in Figure 8), activation in the AON is demonstrates a non-linear relationship with experience. In particular, situations of both extreme unfamiliarity, such as that found in novices, and extreme familiarity, such as that found in CJ, demonstrate greater BOLD activity within action observation regions than actions that are moderately familiar (OTs).

Novices in the PRE condition fall on the left of this proposed model, as they are observing very unfamiliar and novel actions. This is similar to recent findings of greater sensorimotor activation when individuals observe novel or unfamiliar gestures, or unusual robot-like movements compared to human movements (Cross et al., 2011; Liew et al., 2011). All of these situations are novel, interesting, and unfamiliar, and activate one's own sensorimotor regions more perhaps for visuomotor learning by generating a corresponding motor representation for the observed actions. Experienced OTs, and novices after visual experience, may be considered generally or moderately familiar—that is, they have prior experience with the residual limb actions and demonstrate an interest in them, but have neither the novelty nor need for visuomotor learning of novices in the PRE condition, nor the expertise of CJ with his own matching motor repertoire, to generate a significantly increased sensorimotor response to the observed actions. After having seen some residual limbs before, and after having generated a model for these types of effectors, they no longer need or may be as interested in using their own motor regions to simulate the observed action—at least, no more than when observing hand actions. Thus, they fall in the middle, lowest portion of the model.

Completing the other end of the graph, CJ, in many ways, can be thought of as an “expert” in understanding residual limb actions. He not only has extensive visual and real-life experience with individuals who have residual limbs, but he also has first-hand motor experience of residual limb actions. While his limb does not exactly match the residual limb viewed in the stimuli, he still has a greater degree of personal motor experience with a similar type of end effector. Thus, when he sees an individual with residual limbs that are not exactly like his own but share similar kinematics, he may activate his own motor regions more strongly, since he is paying more attention to the nuances of her actions and comparing it to how he might do it himself and activating his own strongly corresponding internal models. Accordingly, he reports being more interested in watching how another individual with residual limbs performs actions than how someone with hands performs them, and he may have a greater motor representation for this type of effector through both visual and personal motor experience. The personal motor experience may also relate to the laterality of his results (as he demonstrated greater left-hemisphere activity than novices or OTs) It should be noted that it is possible that CJ may also have altered cortical representations of body parts due to his own experiences with residual limbs. While there was no sensorimotor activity during HAO compared to residual limb, future studies may confirm the current results by recruiting more individuals that could be classified as having “extreme” experience, such as CJ, with a variety of limb differences.

The current study is limited as the only individual with “extreme” experience has both visual and motor experience with residual limbs. Thus, in the case of CJ, it is unclear whether it is motor or visual experience that is driving the increased AON activity. However, the correlation between prior experience in OTs—who have visual experience but no motor experience with residual limbs—also demonstrates this trend of increased AON activity with increased experience. Thus, it seems that either motor or visual expertise could drive increased activity in the AON. To further investigate the issue of whether extreme motor or visual expertise is responsible for the change, future studies might recruit participants who are family or close friends with individuals with limb differences and who have a lifetime of visual, but not motor, experience with limb differences.

These results resonate with the prior studies of expertise, in which ballet or capoeira dancers observed the dance within their own expertise as well as one that was visually similar but not their expertise (Calvo-Merino et al., 2005). In both situations, the observers are expert in the *type* of movement they are observing, but have not seen these exact stimuli before. Thus, they may attend more to these stimuli because they are interested in how this action, within their realm of expertise, is precisely performed. Similarly, people who watch actions they have practiced extensively may activate their corresponding motor regions more because they are more focused on the nuances of the actions compared to those that they have not learned and have no need to learn (Cross et al., 2009), and they have a more extensive motor representations for these practiced actions, allowing them to evoke a more detailed representation of the observed actions. In addition, observing an action performed by a familiar effector could lead to a more accurate representation of the *goal* of the action, thus increasing AON activity. Applied to the current study, participants who have greater experience with individuals with limb differences (such as very experienced OTs, or CJ) may demonstrate greater AON activity when observing the actress with residual limbs because they are more familiar with the goals of residual limb actions and thus are able to evoke these more detailed representations when observing the actress's actions (Sinigaglia and Rizzolatti, 2011). While the current study did not employ a direct measure of attention or interest/motivation in observing the different clips, a future study may try to quantify interest through explicit means (e.g., questionnaires about how interested participants were in different video clips) or more implicit means (e.g., eye tracking) in order to examine the role of interest during observation of familiar or unfamiliar actions.

In the current experiment, we asked participants to focus on identifying what actions they were observing, rather than specifically calling attention to the way in which the actions were performed. Thus, it is possible that attending to the goal of the actions with an intent to identify them may have resulted in these increased patterns of activation. That is, the increased AON response could be due to an increased processing of the action goal, rather than action kinematics, of the individual with residual limbs compared to the individual with hands. Previous work in primates has shown that mirror neurons from both inferior parietal and premotor (macaque area F5) regions respond to the goal of an action, despite different kinematics (Fogassi et al., 2005; Umiltà et al., 2008). Studies in humans have also shown that the AON is active when observing goal-matched actions performed by two different effectors (hands vs. robots), with no significant differences between different effectors as long as the goal of the action was the same and was possible for the viewer (Gazzola et al., 2007). Thus, it is possible that the AON activity observed in the present study is related to goal representation. However, further investigation is needed to understand what the AON is encoding during novel and familiar actions.

An alternative explanation is that understanding the goal of the action requires more effort when observing novel, compared to familiar, bodies because the AON is learning to pair a familiar goal with an unfamiliar body, or vice-versa. This is in line with a prior study, in which participants were asked explicitly to try to understand and identify symbolic gestures (Liew et al., 2011). These results showed that when participants tried to identify the novel gestures, they also engaged the AON to a greater extent than when identifying familiar gestures. Thus, a basic low-level motor representation may be necessary prior to being able to identify or name the action, and the AON activity may represent either the kinematic representation of the novel action or the pairing of the novel kinematics with a familiar goal.

Finally, the increased activity when observing a novel, compared to familiar, effector could support a predictive encoding account of the AON as put forth by Kilner et al. (2007) and as applied to the results and discussion in Cross et al. (2011). This theory posits that AON activity encodes the prediction error between expected and observed actions. Thus, when observing actions or individuals for which one has less knowledge (e.g., few priors), the prediction error will be larger due to less informed expectations. On the other hand, when an individual has more knowledge (e.g., more priors), the prediction error should be smaller as the expected action should be informed by prior experiences with that action and should thus better match the observed action. This is reflected in our findings, with novices, who have less *a priori* knowledge about residual limb actions compared to hand actions and who show greater AON activation for these novel actions. Over time, as they gain more experience with residual limb actions, their prediction error becomes similar for observation of the residual limb and they may have similar prediction errors for residual limb and hand actions, as evidenced by an attenuated AON signal.

In summary, examining the current data in light of previous findings, we suggest that experience—and likely, related factors such as attention, interest, motivation, task (e.g., motor learning), and novelty—modulate BOLD activity in the one's own sensorimotor regions when observing a wide variety of actions and effectors. Experience may be described along a continuum, from very little experience, to moderate experience, to extreme experience, and the AON activity in response to each of these situations may fit into a non-linear, U-shaped model (Cross et al., 2011) that may also take into account the many factors related to experience, such as attention. Further studies may examine how these complex interactions between real-life contexts and emotions affect activity within the AON, and how other neural networks involved in social cognitive work in tangent with the AON to support experience-driven social understanding.

## Conclusion

Our everyday experiences shape our neural responses to individuals unlike ourselves. The current study demonstrates that real-life interactions with individuals who differ from ourselves attenuates the neural response in our own sensorimotor regions when observing them. In individuals with greater experience, increased experience may in fact engage frontal regions of the AON to a greater extent, possibly to encode the goals, rather than the kinematics, of the observed actions. In addition, a case examination of CJ, an individual with congenital below elbow amputations, demonstrates that extreme visual experience and motor familiarity—particularly for something that is not common in the general population–may allow an individual to activate increased regions of the sensorimotor cortex due to existing motor representations. Altogether, these results support a recently-proposed non-linear model of experience-related modulations on the AON, in which both extreme novelty and extreme expertise have the potential to activate one's own sensorimotor regions than a general, mid-range level of experience or interest. Thus, our own real-life interactions, which may be intertwined with our attention, interests, and motivations, may modulate neural regions that support our ability to understand the actions of others, especially those who are unlike ourselves.

### Conflict of interest statement

The authors declare that the research was conducted in the absence of any commercial or financial relationships that could be construed as a potential conflict of interest.
