# Supplementary material for: Both novelty and expertise increase action observation network activity
Source: Front Hum Neurosci. 2013 Sep 13;7:541. doi: 10.3389/fnhum.2013.00541 (PMC3772553; doi:10.3389/fnhum.2013.00541)

**Supplementary Information**

**Interaction between experience and frontal versus parietal AON regions**

Different components of the AON may perform different roles in action understanding. While novices previously demonstrated strong activation in the parietal component of the AON, associated with generating kinematic representations of actions, we hypothesized that experienced individuals may activate more of the frontal component of the AON, associated with extracting action goals from an observed action. To test this hypothesis, we used a 2x2 mixed measures ANOVA with AON ROI (bilateral IFG/PMv, bilateral IPL) as within subject factors and Experience (novice, experienced OTs) as a between subjects factor.

**Methods:** Region of interest analyses were also performed for *a priori* regions in the AON (bilateral inferior frontal gyrus/ventral premotor cortex and inferior parietal lobules (IFG/PMv and IPL)). These four regions were defined by the overlap between action observation and action execution during the AON localizer run, and further masked by anatomical definitions based on the probabilistic Harvard-Oxford atlas of the IFG/PMv and IPL respectively (results of which are shown in Liew et al., 2013). IFG and ventral premotor regions were combined into one region of interest as prior meta-analyses of the AON suggest that both comprise the frontal component of the AON (Van Overwalle & Baetens, 2009). Percent signal change (%SC) for the observation of each effector (HAO/RLAO) compared to the control still image (HS/RLS) was then extracted using Featquery in FSL and contrasted in paired t-tests. ROI values were used in a 2x2 ANOVA (Experience X Region), which was conducted in SPSS (2009, Chicago, IL, www.spss.com). The factor of Experience compared novices and experienced OTs.

**Results:** There was no main effect of ROI alone in either bilateral or right hemisphere only comparisons (bilateral: F=0.431, p=.53; right hemisphere only: F=.315, p=.58). There was also no main effect of Experience (F=0.310, p=.58). There was a marginally significant interaction between these two (F=3.77,p=.058). When comparing the right hemisphere ROIs only (right IFG/PMv and IPL), the interaction with Experience was greater (F=5.899, p=.023; see *Figure S2*).

**Discussion:** This 2x2 ANOVA of Experience versus Frontal/Parietal AON suggests that novices tended to activate the parietal lobule more when observing the novel effector, while experienced OTs activated the frontal component of the AON more. This finding is not surprising as the parietal and premotor cortices perform vastly different functions: the parietal cortex is primarily associated with encoding the kinematics of an action, while the premotor cortex is primarily associated with the goal and higher-level plan for an action. Thus, while novices may rely more on generating an internal model of a novel action, experienced OTs may rely more on extracting the action goal from the observed action in order to understand the individuals’ higher-level goal. Furthermore, this may occur more on the right hemisphere as the right hemisphere is associated with body schema, body integration, and spatial representation, functions which may be important when observing a body unlike one’s own (Roth, 1949; McGeoch et al., 2011). While further research is necessary to determine how exactly these regions are differentially modulated, these results begin to suggest that not only the amount but also the regions of AON activity are modulated by type and amount of experience.

**Post-scanning Questionnaire Questions**

1. Do you have any experience with people with physical disabilities? (This includes prior visual experience, in movies, on tv shows, in public, etc.)

In particular, do you have any experience with people with limb differences? If so, where was the limb difference and what was the extent of your experience?

2. If so, how often?

3. If so, what type of contact do you generally have?

- Sibling
- Child
- Adult friend
- Parent
- Multiple Contacts
  - Please describe:___________________________________________________
- Other
  - Please describe:___________________________________________________

4. What is your current occupation?

5. Please list any previous occupations that involved contact with people with physical disabilies.

6. How did you feel about the video clips you saw today (general impressions)?

On a scale of 1 to 10, how familiar would you say you are with people with typically developed hands.

1 2 3 4 5 6 7 8 9 10

very unfamiliar very familiar

On a scale of 1 to 10, how comfortable do you feel around people with typically developed hands.

1 2 3 4 5 6 7 8 9 10

very uncomfortable very comfortable

On a scale of 1 to 10, how familiar would you say you are with people with upper arm limb differences.

1 2 3 4 5 6 7 8 9 10

very unfamiliar very familiar

On a scale of 1 to 10, how comfortable do you feel around people with upper arm limb differences.

1 2 3 4 5 6 7 8 9 10

very uncomfortable very comfortable

On a scale of 1 to 10, do you feel that watching these clips made you any more likely to interact with an individual with typically developed hands?

1 2 3 4 5 6 7 8 9 10

very unlikely very likely

8. On a scale of 1 to 10, do you feel that watching these clips made you any more likely to interact with an individual with upper arm limb differences?

1 2 3 4 5 6 7 8 9 10

very unlikely very likely

9. Did watching these clips help you to understand hand actions better?

1 2 3 4 5 6 7 8 9 10

not at all a lot

10. Did watching these clips help you to understand actions made by individuals with limb differences better?

1 2 3 4 5 6 7 8 9 10

not at all a lot

**Supplementary Figures**

**Figure S1. Schematic of experimental procedure.**

**
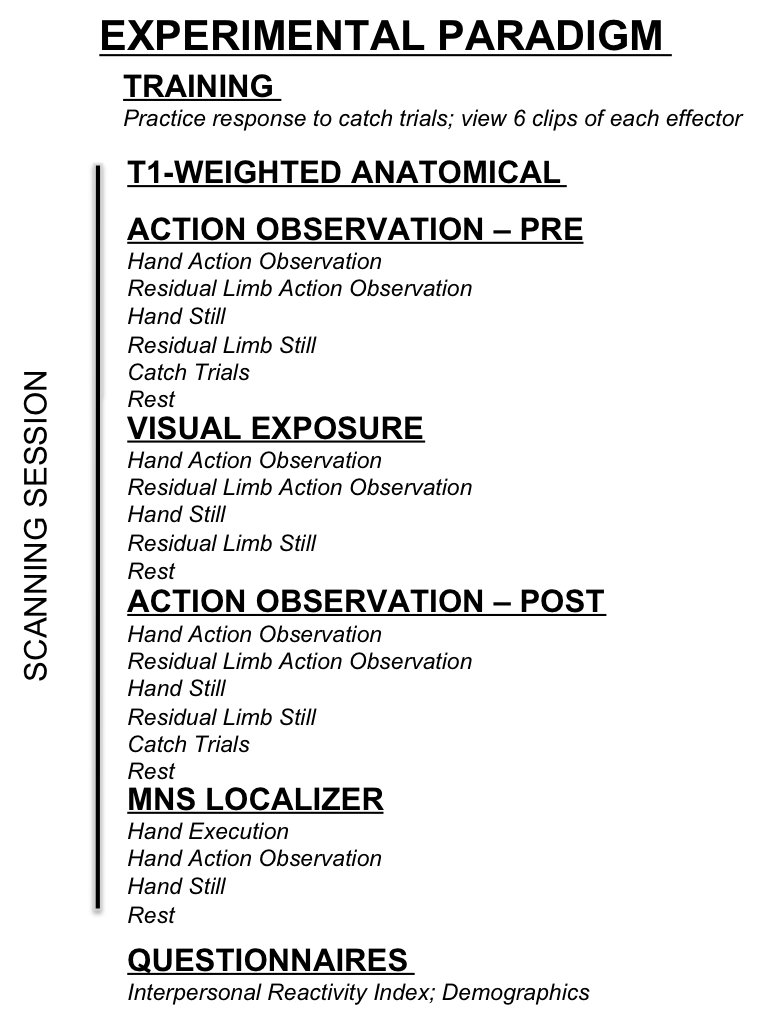
**

**Figure S2. ANOVA between Experience (Novice, Experienced OTs) and AON Region (Frontal, Parietal) during observation of residual limb versus hand actions.** Percent signal change in the right frontal and parietal AON ROIs show a significant interaction with AON region and Experience (experienced OTs versus novices; F=5.899, p=.023).


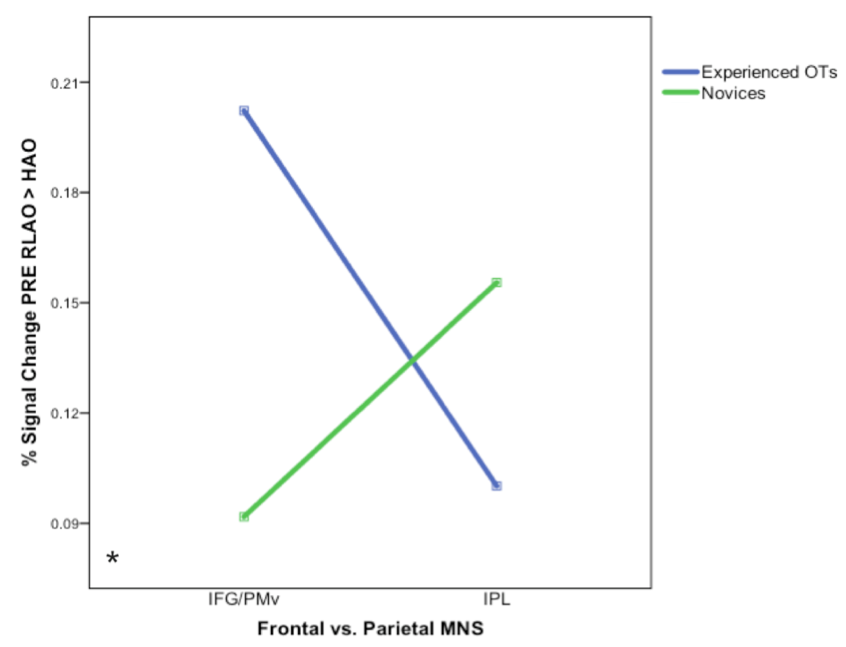

Supplement: Supplementary file 1 [file DataSheet1.DOCX]
